# Zn(C_2_H_4_NO_2_)_2_(SO_2_CH_3_NH_2_)_2_ and Zn(C_2_H_4_NO_2_)_2_[SO_2_(NH_2_)_2_]: Regulation of Unique ZnO(C_2_H_4_NO_2_)_2_ Units by Polar Tetrahedrons for Balanced UV Nonlinear Optical Performance

**DOI:** 10.1002/advs.75567

**Published:** 2026-05-08

**Authors:** Chenjie Cui, Yuchen Yan, Xin Wen, Jindong Chen, Jiyang Wang, Zhanggui Hu, Ning Ye, Guang Peng

**Affiliations:** ^1^ State Key Laboratory of Crystal Materials Tianjin Key Laboratory of Functional Crystal Materials Institute of Functional Crystal Tianjin University of Technology Tianjin China; ^2^ State Key Laboratory of Crystal Materials and Institute of Crystal Materials Shandong University Jinan China

**Keywords:** birefringence, glycine, nonlinear optical, short‐wave ultraviolet, sulfamide

## Abstract

Short‐wave ultraviolet (200–280 nm) nonlinear optical (NLO) crystals are scarce. Herein, by mixing two types of hetero‐coordinate substituted groups, we synthesized two new crystals, Zn(C_2_H_4_NO_2_)_2_(SO_2_CH_3_NH_2_)_2_ (ZGlyM) and Zn(C_2_H_4_NO_2_)_2_[SO_2_(NH_2_)_2_] (ZGlyS), featuring a unique ZnO(C_2_H_4_NO_2_)_2_ secondary functional building unit which tends to assemble into a polar chain. Interestingly, disorder in ZGlyM results in a centrosymmetric structure, whereas in ZGlyS, the replacement of the polar tetrahedral group eliminates disorder and preserves a non‐centrosymmetric structure. Notably, the ZGlyS exhibits a rare balance between a short UV cutoff edge of 209 nm, a large second harmonic generation effect of 1.36 × KH_2_PO_4_, and a large birefringence of 0.126@546 nm. Remarkably, its phase‐matching range covers the entire transparent wavelength range, namely, the shortest phase‐matching wavelength is 209 nm. This work provides a potential short‐wave UV NLO crystal and offers new insights for the design of such crystals.

## Graphical abstract

The modulation of polar tetrahedra enabled the synthesis of an outstanding short‐wave UV NLO crystal, Zn(C_2_H_4_NO_2_)_2_[SO_2_(NH_2_)_2_], which achieves a rare balance of a large bandgap (5.93 eV), high birefringence (Δn = 0.126 at 546 nm), and strong SHG response (1.36 × KDP), especially achieving excellent phase‐matching throughout the full transparency range.

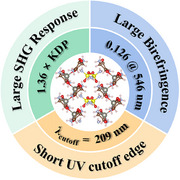

## 1 Introduction

Nonlinear optical (NLO) crystals are core components in all‐solid‐state lasers due to their unique laser frequency conversion capabilities. The development of such a type of optoelectronic functional crystals has greatly advanced the field of all‐solid‐state lasers, and they have been widely applied in various fields such as medical, biological, and semiconductor materials [1, 2, 3, 4, 5]. The primary requirement for NLO crystals is that they must crystallize in non‐centrosymmetric (NCS) space groups, which contrasts with the natural tendency of crystals to crystallize in low‐energy centrosymmetric (CS) space groups. Currently, the demand for ultraviolet (UV) NLO crystals mainly focuses on the short‐wave UV region (200–280 nm) and even shorter deep‐UV region. Outstanding UV NLO crystals need to possess the shortest possible UV cutoff edge (λ_UV_) to meet transmission requirements, large second harmonic generation (SHG) effects to enhance conversion efficiency (usually ≥ 1.0 × KH_2_PO_4_ (KDP)), and sufficient birefringence (∆n, usually ≥ 0.06) for efficient phase‐matching capabilities. However, achieving a balance among the three types of performance has always been a highly challenging task in this field [6, 7, 8, 9, 10].

The anionic group theory emphasizes the decisive role of the microscopic functional units and their spatial arrangement in determining the above core properties of NLO crystals [11]. Planar triangular π‐conjugated groups and tetrahedral groups are two typical microscopic functional units for exploring UV NLO crystals. For a long time, the exploration of UV NLO crystals has mainly focused on the borates, as planar triangular [BO_3_]^3−^ and polymeric [B_3_O_6_]^3−^ six‐membered ring groups are π‐conjugated planar groups that enable crystals to possess relatively large SHG effects and birefringence [5, 10, 11, 12]. In recent years, similar planar π‐conjugated groups, such as [CO_3_]^2−^, [C(NH_2_)_3_]^+^ and cyanuric acid groups [H_3‐x_C_3_N_3_O_3_]^x−^ (x = 0, 1, 2, 3), have been widely applied in the exploration of UV NLO crystals, yielding significant achievements [13, 14, 15, 16, 17, 18, 19, 20]. In contrast, tetrahedral groups like MO_4_ (M═B, P, S, Si) usually lead to weak second‐order NLO coefficients and low optical anisotropy due to their isotropic structural characteristics, even though they can achieve larger band gaps. To address this issue, the tetrahedra‐modification strategy *v* *i* *a* hetero‐coordination atom substitution has been widely employed. Polar tetrahedral groups such as [BO_x_F_4‐x_]^(x+1)−^, [SO_3_F]^−^, [SO_3_NH_2_]^−^, SO_2_(NH_2_)_2_ have been screened out, which have been successfully applied to synthesize several outstanding UV and deep‐UV NLO crystals, including NH_4_B_4_O_6_F, (NH_4_)_2_PO_3_F, NaNH_4_PO_3_F·H_2_O, C(NH_2_)_3_SO_3_F, M(NH_2_SO_3_)_2_ (M═Sr, Ba), [SO_2_(NH_2_)_2_], and others [18, 21, 22, 23, 24, 25]. Similarly, planar π‐conjugated groups can also be further expanded into new functional units *v* *i* *a* the same substitution strategies. Meanwhile, the enhanced polarity and reduced symmetry may more favorably facilitate the formation of NCS space groups.

In addition to the choice of functional units, their spatial arrangement is also crucial for NLO crystals. It not only determines the symmetry of the crystal but also dictates its core properties. In particular, the parallel alignment of groups at the microscopic level is most beneficial for enhancing NLO coefficients and birefringence [12]. However, CS arrangements of groups are almost catastrophic for NLO effects. For instance, the reverse parallel arrangement of two rigid planar triangular groups directly cancels out the NLO effects. This is a common and unavoidable problem in the exploration of NLO crystals.

Guided by the above insights, we have selected two types of groups with large band gaps as the basic functional building units (FBUs) for designing NLO crystals, namely, glycine (Gly) and polar tetrahedral groups, including methanesulfonamide SO_2_NH_2_CH_3_ and sulfamide SO_2_(NH_2_)_2_. The Gly group can be seen as a hetero‐coordinated substituted derivative of the typical UV FBU [CO_3_]^2−^ group, where an oxygen atom is replaced by an aminomethyl ‐CH_2_NH_2_. By mixing the two types of polarity‐enhanced groups, two new crystals, Zn(C_2_H_4_NO_2_)_2_(SO_2_CH_3_NH_2_)_2_ (ZGlyM) and Zn(C_2_H_4_NO_2_)_2_[SO_2_(NH_2_)_2_] (ZGlyS), were successfully synthesized. Interestingly, both crystals feature unique T‐shaped ZnO(C_2_H_4_NO_2_)_2_ secondary FBUs. However, different polar tetrahedral units drive the T‐shaped units from a disordered to an ordered arrangement, thereby transforming the crystal structure from CS to NCS. More importantly, the ZGlyS crystal achieves a rare balance of core properties, including a short UV cutoff edge of 209 nm, a large SHG coefficient of 1.36 × KDP, and significant birefringence of 0.126@546 nm. Notably, the shortest phase‐matching wavelength of this crystal can reach 209 nm, which means that it is a rare crystal that can achieve phase matching across the entire transparent wavelength range [16]. This work not only provides a potential alternative material for short‐wave UV NLO crystals but also offers new insights for the design of NCS crystals owing to its intriguing structural features.

## 2 Results and Discussion

**FIGURE 1:**
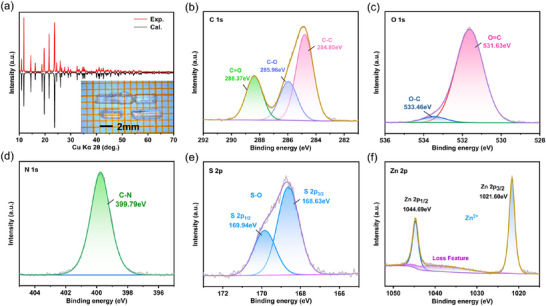
a) Experimental and simulated powder X‐ray diffraction (XRD) patterns. inset: crystal photograph; b‐f) X‐ray photoelectron spectroscopy (XPS) spectra of ZGlyS.

Both crystals were synthesized by the solution evaporation method. The single crystal of ZGlyS was obtained by using Glycine, SO_2_(NH_2_)_2_, and ZnCO_3_ as raw materials. As for ZGlyM, the raw materials are Glycine, SO_2_CH_3_NH_2_, ZnF_2_. Detailed experimental details can be found in the Supporting Information. It is worth noting that the synthesis of ZGlyM is very challenging, with an extremely low yield. Due to low yield and the inability to obtain sufficient ZGlyM, this study primarily focuses on characterizing the performance of ZGlyS crystals. Powder X‐ray diffraction (XRD) studies confirmed the purity of the polycrystalline sample (Figure 1a). The scanning electron microscope elemental mapping (Figure S1) confirmed the presence of Zn, C, O, N, and S in the ZGlyS single crystal, and X‐ray photoelectron spectroscopy (XPS) simultaneously verified these five elements (Figure 1b–f and Figure S2). The characteristic peaks of Zn^2+^ at 1044.69 and 1021.60 eV correspond to Zn 2p_1/2_ and Zn 2p_3/2_, respectively. The characteristic peaks of S^6+^ at 169.94 and 168.63 eV correspond to S 2p_1/2_ and S 2p_3/2_, respectively. The characteristic peak at 399.79 eV in N 1s can be attributed to the C‐N bond. The characteristic peaks at 533.46 and 531.63 eV in O 1s correspond to the O─C bond and the O═C bond in O^2−^, respectively. The characteristic peaks at 288.37, 285.96, and 284.80 eV in C 1s correspond to the C═O bond, C─O bond, and C─C bond, respectively. As the infrared (IR) spectrum (Figure S3) of ZGlyS shows, a strong band around 3196 cm^−1^ is attributed to N─H stretching, while the C═O stretching vibration appears at 1580 cm^−1^. C─H bending vibrations are observed at 1447 and 730 cm^−1^, and the bending vibration of ‐NH_2_ is detected at 1380 cm^−1^. C─N stretching vibrations occur at 1330, 1270, and 1100 cm^−1^, whereas the S─O stretching vibration is observed at 1046 cm^−1^. The C─C stretching vibration appears at 972 cm^−1^, and a strong absorption at 531 cm^−1^ corresponds to the bending vibration of the Zn─O bond. These spectral features collectively confirm the presence of the functional groups in the compound. In addition, the TG‐DSC analysis curve (Figure S4) indicates that the ZGlyS crystal can maintain thermal stability up to approximately 180°C. A distinct endothermic peak appears at 187°C. Subsequently, as the temperature further increases, the TG curve shows significant mass loss.

**FIGURE 2:**
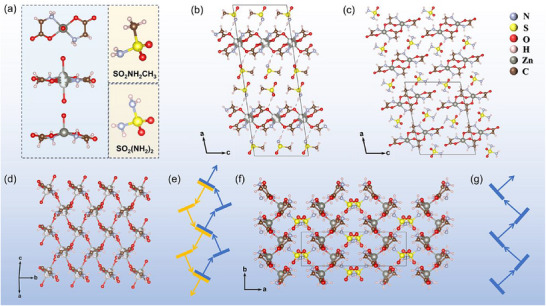
Crystal structures. a) ZnO(C_2_H_4_NO_2_)_2_, SO_2_NH_2_CH_3_, and SO_2_(NH_2_)_2_ groups; Crystal structures of (b) ZGlyM and (c) ZGlyS; d‐g) The arrangement of ZnO(C_2_H_4_NO_2_)_2_ in ZGlyM and ZGlyS and their simplified schematic diagram.

The crystal structures of ZGlyM and ZGlyS were determined by the single‐crystal XRD analysis. These two crystals crystallize in the space group of *C*2/*c* and *C*2, respectively (detailed crystallographic data are listed in Tables S1–S4 in the Supporting Information). Figure 2a–c shows their main FBUs and main structures. In both crystals, two Gly groups and one Zn^2+^ tend to form a unique bis‐ligand bidentate chelated ZnO(C_2_H_4_NO_2_)_2_ secondary FBU. In both crystals, the SO_2_NH_2_CH_3_ and SO_2_(NH_2_)_2_ groups are spatially isolated from the secondary FBUs, yet they are interconnected through hydrogen bonds and intermolecular interactions. A notable difference is that in ZGlyM, the position of Zn atoms within the ZnO(C_2_H_4_NO_2_)_2_ is disordered, resulting in centrosymmetric structural features due to statistical distribution. In contrast, in ZGlyS, this secondary FBU exhibits no disorder and displays a pronounced polarity. It is noteworthy that this secondary FBU would normally present a T‐shaped polar unit, although its Gly groups exhibit an undesired antiparallel arrangement. In addition, these secondary FBUs tend to form zigzag polar chains by sharing oxygen atoms at their ends. As shown in Figure 2d–g, disorder in ZGlyM causes the polar zigzag chains to flip mirror‐symmetrically, creating a centrosymmetric structure. In contrast, in ZGlyS, changes in the polar tetrahedral groups eliminate disorder, preserve the ordered arrangement of the chains, and convert the crystal space group from CS to NCS. Notably, the polar chains along the *b*‐axis exhibit enhanced polarity, and the interstitial SO_2_(NH_2_)_2_ groups display the same trend. To verify this point, the dipole moments of ZnO_3_N_2_ and SO_2_N_2_ groups within a minimum repetition unit of ZGlyS were calculated. The calculated direction and magnitude of the dipole moment of ZnO_3_N_2_ and SO_2_N_2_ groups have been listed in Table S5. The local dipole moments of the four individual ZnO_3_N_2_ and SO_2_N_2_ groups are all 1.68 and ∼4.7 D (D = Debye), respectively [26, 27, 28]. The *x* and *z* components of the polarization from the four ZnO_3_N_2_ and SO_2_N_2_ groups within a unit cell cancel each other completely, whereas their *y* components, enhanced due to the constructive addition of polarities, contribute to a net dipole moment of 3.31 D and 3.87 D, respectively. This indicates that the polarizations of both groups undergo constructive addition along the y‐axis, contributing synergistically to their SHG effects.

**FIGURE 3:**
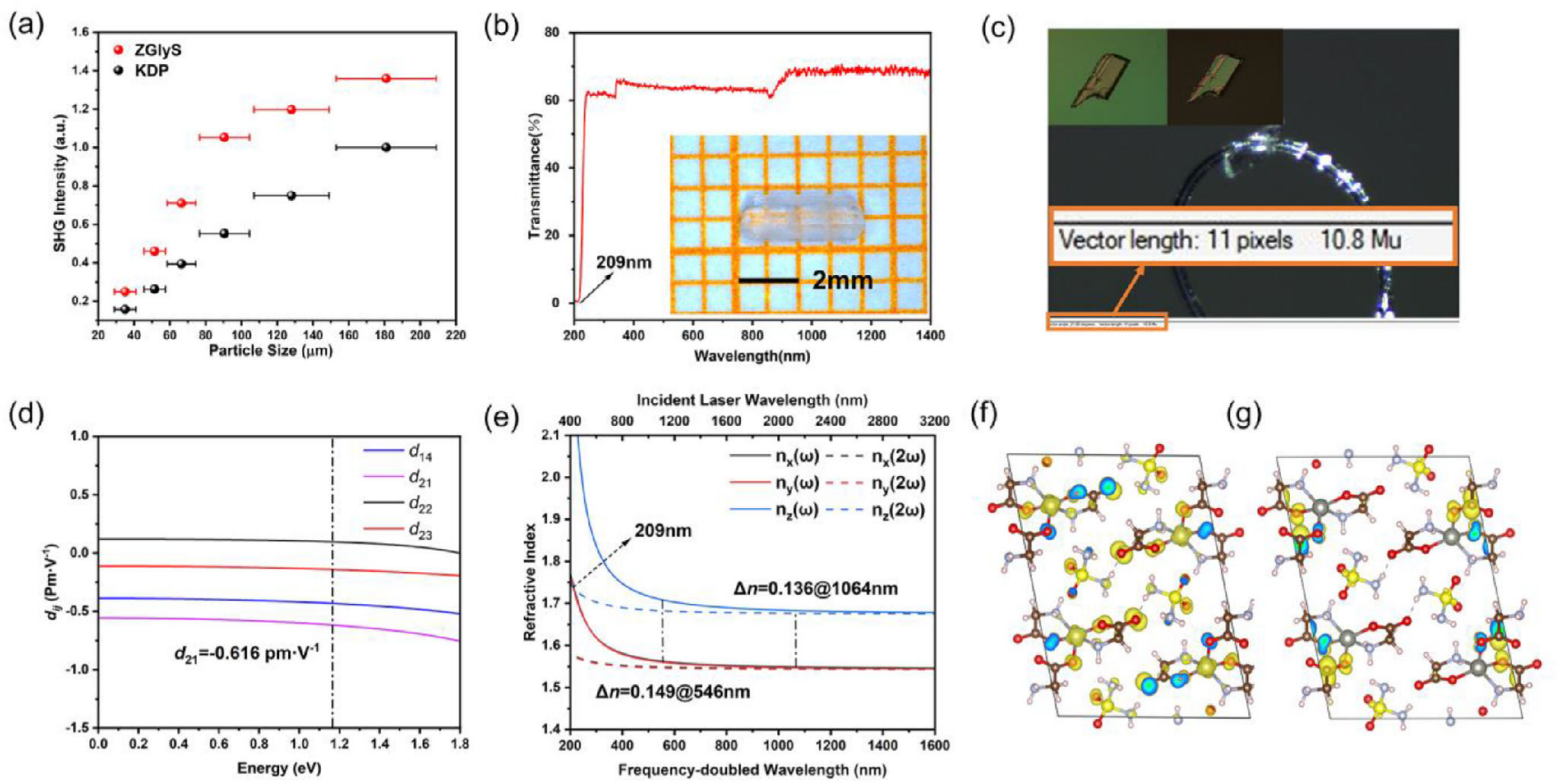
The optical properties of ZGlyS. a) The powder SHG measurements with KDP as a reference; b) The ultraviolet‐visible‐near infrared transmittance spectrum. inset: crystal photograph; c) Birefringence measurements; d) Calculated NLO coefficient; e) Calculated refractive indices; f, g) SHG‐weighted electron density maps of the occupied and unoccupied states in the VE process.

The polycrystalline SHG effect of ZGlyS was measured using the Kurtz−Perry method with a Q‐switched Nd: YAG solid‐state laser at a wavelength of 1064 nm [29]. As shown in Figure 3a, it not only realized a large SHG effect of 1.36 × KDP within the 150–212 µm particle size range, corresponding to an effective NLO coefficient of approximately 0.53 pm·V^−1^ according to the 0.39 pm·V^−1^ of KDP, but also exhibited phase‐matching behavior.

The ultraviolet‐visible‐near‐infrared (UV–vis–NIR) transmission spectrum (Figure 3b) shows that ZGlyS possesses a high transmittance between 240–1400 nm, with a short UV cutoff edge of 209 nm, corresponding to a large bandgap of 5.93 eV. Its excellent short‐wave UV transparency means that the crystal has potential applications in the short‐wave UV spectral region.

By using a polarizing microscope with a 546 nm light source, the birefringence of ZGlyS was preliminarily measured [30]. The formula for calculating the birefringence can be described using the following formula: R = Δ*n* × T, where R, Δ*n*, and T represent the optical path difference, birefringence, and the thickness of the sample. As shown in Figure 3c, the R of ZGlyS is 1365 nm with a thickness of 10.8 µm. The corresponding birefringence was calculated to be 0.126@546 nm. Such a large birefringence is comparable with the well‐known commercial birefringent crystals *α*‐BaB_2_O_4_ (0.122 @ 532 nm) [31]. It indicates that the compound is a promising candidate for a short‐wave UV large‐birefringence crystal material.

To better understand the microcosmic mechanism of optical properties, the density functional theory (DFT) calculations for ZGlyS were performed [32, 33]. The calculated indirect band gap was 4.689 eV (Figure S5), which is underestimated compared to the experimental value. This is mainly due to the inherent properties of the generalized gradient approximation (GGA) as the exchange‐correlation functional. Therefore, the bandgap was corrected to the experimental value using a 1.241 eV scissors operator to improve the accuracy of optical property calculations [34, 35]. Because of the point group 2, under the constraints of the Kleinman symmetry, ZGlyS possesses four independent, nonzero NLO coefficients, *d*_14_, *d*_21_, *d*_22_, and *d*_23_. The calculated frequency‐dependent NLO coefficients were plotted in Figure 3d. The values at 1064 nm are *d*_14_ = −0.491 pm·V^−^^1^, *d*_21_ = −0.616 pm·V^−^^1^, *d*_22_ = 0.096 pm·V^−^^1^, and *d*_23_ = −0.140 pm·V^−^^1^, respectively. The largest *d*_21_ is approximately 1.58 × KDP, which is close to the experimental value. The slightly lower experimental SHG intensity is attributed to random crystal orientation during measurement. In addition, the calculated refractive index curve of ZGlyS shows a strong optical anisotropy, with n_z_ > n_x_ ≈ n_y_ (Figure 3e). The theoretically calculated birefringence at wavelengths of 546 and 1064 nm is 0.149 and 0.136, respectively, which matches well with the experimental data, thereby supporting the reliability of the experimental results. The experimentally measured birefringence is slightly lower than the theoretical value, which may arise from errors in crystal thickness and the visual determination of the optical path difference during measurement. Its suitable birefringence enables excellent phase‐matching capabilities. Based on the calculated refractive indices, the shortest type‐I phase‐matching wavelength is 209 nm, which is as short as its UV cutoff edge. Such phase‐matching across the entire transparency range is rare, indicating that ZGlyS is a promising short‐wave UV NLO and birefringent material.

The total and partial density of states (DOS and PDOS, Figure S6) plots of ZGlyS show that the top of the valence band (VB) is mainly dominated by the O‐2*p* and Zn‐3*d* orbitals, with the C‐2*p* and N‐2*p* orbitals making secondary contributions. The bottom of the conduction band (CB) is significantly contributed to by the C‐2*p*, O‐2*p*, and Zn‐4*s* orbitals. It is known that the optical properties of the crystal are primarily influenced by the electronic behavior near the Fermi level. Therefore, it can be inferred that the optical performance of ZGlyS is mainly determined by Gly and ZnO_2_N_2_, with the SO_2_(NH_2_)_2_ groups making secondary contributions. The three types of FBUs synergistically determined the optical properties of ZGlyS. Because the SHG‐weighted densities of the occupied and unoccupied states in the virtual electron (VE) process make dominant contributions to the SHG effect [36, 37], the SHG‐weighted density of the largest tensor *d_21_* was calculated to intuitively verify the origin of the SHG effect. Figure 3f,g clearly show that the carboxyl group on Gly, ZnO_2_N_2_, and SO_2_(NH_2_)_2_ groups all exhibit obvious electronic contributions to the SHG effect, with the former two contributing most prominently. This further supports the above conclusions.

**FIGURE 4:**
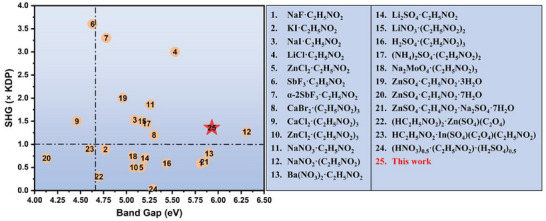
Comparison of SHG intensities and band gaps between the ZGlyS crystal and the main members of the glycine‐containing family.

Compared with the main members of the Gly‐containing family (Figure 4, Table S6) [38, 39, 40, 41, 42, 43, 44, 45, 46, 47, 48, 49, 50, 51, 52, 53, 54, 55, 56, 57, 58, 59, 60], the ZGlyS crystal stands out as an exceptional new representative. It not only features a novel structure but also successfully addresses the challenging balance between a large bandgap, high birefringence, and strong SHG response, especially achieving phase‐matching across the entire transparency range. It possesses a comparatively large bandgap, making it one of the few members approaching 6.0 eV. It is worth noting that although the band gap of crystal #12 appears to be larger, nitrite exhibits an intrinsic absorption peak around 350 nm. For example, the λ_UV_ of well‐known NaNO_2_ is at 350 nm. The SHG intensity of ZGlyS is not the strongest among all members, but its performance is particularly notable in the short‐wave region. In addition, ZGlyS is one of the crystals with the largest birefringence. Its birefringence significantly surpasses most others with reported birefringence, such as (HC_2_H_5_NO_2_)_2_·Zn(SO_4_)(C_2_O_4_) (0.03@1064 nm), HC_2_H_5_NO_2_·In(SO_4_)(C_2_O_4_)(C_2_H_5_NO_2_) (0.02@1064 nm), SbF_3_·C_2_H_5_NO_2_ (0.057@1064 nm), and *β*‐2SbF_3_·C_2_H_5_NO_2_ (0.07@546 nm) [43, 44, 59]. Meanwhile, it is comparable to the top performers in this family, including *α*‐2SbF_3_·C_2_H_5_NO_2_ (0.146@1064 nm) and Li_2_SO_4_·C_2_H_5_NO_2_ (0.144@550 nm) [44, 51]. Although sufficient data are not yet available to directly compare their phase‐matching capabilities, the excellent performance of ZGlyS in achieving phase matching across the entire wide transparency range undoubtedly positions it as one of the outstanding members in this family.

## 3 Conclusion

In summary, by mixing Gly and different polar tetrahedra, two new crystals, Zn(C_2_H_4_NO_2_)_2_(SO_2_CH_3_NH_2_)_2_ (ZGlyM) and Zn(C_2_H_4_NO_2_)_2_[SO_2_(NH_2_)_2_] (ZGlyS), featuring a unique ZnO(C_2_H_4_NO_2_)_2_ secondary unit which tends to assemble into a polar chain, were designed and synthesized. It demonstrates the structural regulation ability of polar tetrahedral units from disordered/centrosymmetric to ordered/noncentrosymmetric. Remarkably, ZGlyS achieves a rare balance of a large bandgap (5.93 eV), high birefringence (Δn = 0.126 at 546 nm), and strong SHG response (1.36 × KDP), especially achieving excellent phase‐matching throughout the full transparency range, with a shortest phase‐matching wavelength of 209 nm. These properties highlight its promise as a shortwave UV nonlinear optical crystal.

## Conflicts of Interest

The authors declare no conflict of interest.

## Supporting information




**Supporting File**: advs75567‐sup‐0001‐SuppMat.docx.

## Data Availability

Deposition Numbers 2540952 (ZGlyM) and 2540950 (ZGlyS) contain the supplementary crystallographic data for this paper. These data can be obtained free of charge by the joint Cambridge Crystallographic Data Centre and Fachinformationszentrum Karlsruhe Access Structure service.
