# Supplementary material for: Zn(C2H4NO2)2(SO2CH3NH2)2 and Zn(C2H4NO2)2[SO2(NH2)2]: Regulation of Unique ZnO(C2H4NO2)2 Units by Polar Tetrahedrons for Balanced UV Nonlinear Optical Performance
Source: Adv Sci (Weinh). 2026 May 8;13(43):e75567. doi: 10.1002/advs.75567 (PMC13336050; doi:10.1002/advs.75567)
Supplement: Supplementary file 1 — Supporting File: advs75567‐sup‐0001‐SuppMat.docx. [file ADVS-13-e75567-s001.docx]

**Supporting Information**

**Zn(C_2_H_4_NO_2_)_2_(SO_2_CH_3_NH_2_)_2_ and** **Zn(C_2_H_4_NO_2_)_2_[SO_2_(NH_2_)_2_]: Regulation of Unique ZnO(C_2_H_4_NO_2_)_2_ Units by Polar Tetrahedrons for Balanced UV Nonlinear Optical Performance**

Chenjie Cui,^[a],#^ Yuchen Yan,^[a],#^ Xin Wen,^[b]^ Jindong Chen,^[a]^ Jiyang Wang,^[a,b]^ Zhanggui Hu,^[a]^ Ning Ye,*^[a]^ Guang Peng*^[a]^

[a] State Key Laboratory of Crystal Materials, Tianjin Key Laboratory of Functional Crystal Materials, Institute of Functional Crystal, Tianjin University of Technology, Tianjin 300384, China

[b] State Key Laboratory of Crystal Materials and Institute of Crystal Materials, Shandong University, Jinan 250100, China

# These authors contributed equally to this work.

* nye@email.tjut.edu.cn (Ning Ye); gpeng@email.tjut.edu.cn (Guang Peng)

**Reagents**

Glycine (Adamas, 99.9%), SO_2_(NH_2_)_2_ (Adamas, 98%), ZnCO_3_ (Adamas, ≥57% ZnO), SO_2_CH_3_NH_2_ (Adamas, 98%) and ZnF_2_ (Aladdin, 99%)

**Synthesis**

**Zn(C_2_H_4_NO_2_)_2_(SO_2_CH_3_NH_2_)_2_**

Glycine (0.005 mol, 0.375 g), SO_2_CH_3_NH_2_ (0.005 mol, 0.475 g), and ZnF_2_ (0.0025 mol, 0.258 g) were dissolved in 25 mL of deionized water. The mixture was continuously stirred at room temperature for 4 hours until a clear solution was obtained. The resulting solution was then allowed to evaporate slowly at 35 ℃, during which block-shaped crystals formed at the bottom of the beaker. However, the yield of these crystals was very low.

**Zn(C_2_H_4_NO_2_)_2_[SO_2_(NH_2_)_2_]**

Dissolved stoichiometric Glycine (0.005mol,0.375g), SO_2_(NH_2_)_2_ (0.005mol,0.480g), and ZnCO_3_ (0.005mol,0.627g) in 25 mL of deionized water at room temperature. Continuously stir the mixture for 4 hours to obtain a homogeneous saturated solution. The saturated solution is filtered using filter paper. The filtered solution is slowly evaporated at 60 °C, and after 3-4 days, the solution becomes supersaturated, and needle-shaped crystals are found in the beaker. The crystal yield exceeds 90%.

**Single crystal X-ray diffraction**

The diffraction data were collected at room temperature on a Bruker D8 VENTUE CCD diffractometer and Mo Kα radiation (λ=0.71073 Å). The data was integrated by APEX Ⅲ, and the multi-scan method was used for the absorption corrections. The crystal structures were determined by the direct methods and refined by full-matrix least-squares fitting on *F^2^* using the SHELXS crystallographic software package on the Olex2 program.^[1]^ All atoms except H were refined with anisotropic displacement parameters. The structure was checked by PLATON, and no higher symmetry was found.^[2]^

**Powder X-ray diffraction**

The Powder X-ray diffraction (XRD) patterns data were collected from 10° to 70° (2*θ*) with a step width of 0.01° and a step time of 0.2 s on a SmartLab9KW powder X-ray diffractometer with Cu-Kα radiation (λ = 1.5418 Å).

**Thermogravimetric Analysis and Differential Scanning Calorimetry**

The thermal behavior of ZGlyS was investigated using a METTLER TOLEDO TGA/DSC-1 thermal analyzer under a nitrogen atmosphere. Approximately 7 mg of sample was placed in an Al_2_O_3_ crucible, and the measurement was carried out over the temperature range of 30-800 ^o^C.

**Transmittance Spectroscopy**

UV-vis-NIR transmission spectrum was collected on a UH4150 spectrophotometer.
**Infrared (IR) Spectrum**

The IR spectrum was recorded on a Frontier Mid-IR FTIR spectrometer from 400 to 4000 cm^-1^ at room temperature.

**Second Harmonic Generation (SHG)**

Powder SHG responses were measured with the Kurtz-Perry method using a Q-switched Nd: YAG solid-state laser with a wavelength of 1064 nm.^[3]^ Polycrystalline samples were ground and sieved into several distinct particle size ranges of 25-45, 45-58, 58-75, 75-106, 106-150, 150-212 μm, and then pressed into the container with a thickness of 1mm and diameter of 8 mm. Polycrystalline KH_2_PO_4_ (KDP) was prepared with the same size range as a reference.

**Birefringence**

The birefringence of a ZGlyS crystal at 546 nm was measured on a Nikon Eclipse E200MV POL polarizing microscope. The thickness of the crystal was measured on a Bruker D8 VENTUE CCD diffractometer. The birefringence was determined using the formula: *R = ∆n* × *d*, where *∆n*, *R*, and *d* represent birefringence, optical path difference, and crystal thickness, respectively.^[4]^

**The First-principles Calculation**

The electronic structure calculations were performed by first-principles calculations in the CASTEP package.^[5,6]^ The gradient generalized approximation (GGA) with the Perdew-Burke-Ernzerhof (PBE)^[7]^ functional was adopted to describe the exchange-correlation energy. The optimized norm-conserving pseudopotentials^[7]^ in the Kleinman-Bylander^[8]^ form for all the elements were used to model the effective interaction between atom cores and valence electrons. The valence electrons were treated as Zn: 3*d*^10^4*s*^2^, N: 2*s*^2^2*p*^3^, C: 2*s*^2^2*p*^2^, O: 2*s*^2^2*p*^4^, H: 1*s*^1^, S:3*s*^2^3*p*^4^. The high kinetic energy cutoff of 750 eV and dense 1×3×1 Monkhorst-Pack k-point meshes in the Brillouin zones were chosen. Therefore, a scissor of 1.241 eV was applied to adjust the theoretical band gap to accurately calculate the optical properties of ZGlyS.^[9-10]^

**Table S1.** Crystal data and structure refinement for ZGlyM and ZGlyS.

| Empirical formula | Zn(C_2_H_4_NO_2_)_2_(SO_2_CH_3_NH_2_)_2_ | Zn(C_2_H_4_NO_2_)_2_[SO_2_(NH_2_)_2_] |
| --- | --- | --- |
| Formula weight | 403.73 | 309.61 |
| Temperature[K] | 273.15 | 273.15 |
| *λ*(Å) | 0.71073 | 0.71073 |
| Crystal system | Monoclinic | Monoclinic |
| space group | *C*2/*c* | *C*2 |
| *a* [Å] | 27.9764(15) | 16.5662(5) |
| *b* [Å] | 5.3095(3) | 5.22320(10) |
| *c* [Å] | 9.9232(5) | 12.4901(4) |
| *α*[°] | 90 | 90 |
| *β* [°] | 98.236(2) | 102.0450(10) |
| *γ* [°] | 90 | 90 |
| Volume [Å^3^] | 1458.80(14) | 1056.96(5) |
| *Z* | 4 | 4 |
| *ρ*_calc_ [gcm^−3^] | 1.838 | 1.946 |
| *μ* [mm^−1^] | 2.013 | 2.543 |
| *F*(000) | 832 | 632 |
| θ range [°] | 2.943 to 27.579 | 2.514 to 27.535 |
| Index ranges | -36 ≤ h ≤36 | -21 ≤ h ≤ 21 |
|  | -6 ≤ k≤ 6 | -6 ≤ k ≤ 6 |
|  | -12 ≤ l ≤ 12 | -16 ≤ l ≤ 16 |
| *R*_int_ | 0.0371 | 0.0288 |
| Completeness to *θ* = 25.242 | 99.7 % | 99.3 % |
| Data/Restraints/ Parameters | 1674/2/117 | 2390/1/162 |
| Goodness-of-fit on *F*^2^ | 1.065 | 0.912 |
| Final *R* indexes | *R*_1_ = 0.0321 | *R*_1_ = 0.0240 |
| [*I*≥2σ(*I*)] | *wR*_2_ = 0.0806 | *wR*_2_ = 0.0475 |
| Final *R* indexes | *R*_1_ = 0.0442 | *R*_1_ = 0.0276 |
| [all data] | *wR*_2_ = 0.0864 | *wR*_2_ = 0.0490 |
| Absolute structure parameter | / | 0.026(9) |

**Table S2.** Atomic coordinates and *U*_eq_ [Å^2^ ] for ZGlyM and ZGlyS. *U*_eq_ is defined as 1/3 of the trace of the orthogonalized *U_ij_* tensor.

Zn(C_2_H_4_NO_2_)_2_(SO_2_CH_3_NH_2_)_2_

| **Atom** | ***x*** | ***y*** | ***z*** | ***U*_eq_** |
| --- | --- | --- | --- | --- |
| Zn(1) | 2500 | 2500 | 0 | 31(1) |
| C(2) | 2980(1) | 4306(4) | 2522(2) | 33(1) |
| N(2) | 3046(1) | 556(3) | 1063(2) | 31(1) |
| Zn(2) | 2401(2) | 2150(8) | 243(5) | 31(1) |
| C(3) | 3250(1) | 1900(4) | 2311(2) | 34(1) |
| O(3) | 3089(1) | 5405(3) | 3647(2) | 52(1) |
| O(4) | 2673(1) | 5089(3) | 1578(2) | 46(1) |
| N(1) | 4040(1) | 4879(4) | 179(2) | 46(1) |
| C(1) | 4455(1) | 2357(5) | -1728(2) | 49(1) |
| O(1) | 4803(1) | 2636(3) | 835(2) | 58(1) |
| S(1) | 4352(1) | 2430(1) | -34(1) | 32(1) |
| O(2) | 4048(1) | 320(3) | 159(2) | 40(1) |

Zn(C_2_H_4_NO_2_)_2_[SO_2_(NH_2_)_2_]

| **Atom** | ***x*** | ***y*** | ***z*** | ***U*_eq_** |
| --- | --- | --- | --- | --- |
| Zn(1) | 6936(1) | 2683(1) | 8245(1) | 26(1) |
| O(3) | 7845(1) | 5120(4) | 10435(2) | 28(1) |
| O(4) | 7928(1) | 5044(4) | 8677(2) | 28(1) |
| O(5) | 6473(2) | 4517(4) | 6769(2) | 30(1) |
| O(6) | 6272(2) | 3986(5) | 4963(2) | 43(1) |
| N(3) | 8933(2) | 9001(6) | 10932(2) | 32(1) |
| N(4) | 7314(2) | 75(5) | 7206(2) | 29(1) |
| C(1) | 8844(2) | 7775(9) | 9856(2) | 32(1) |
| C(2) | 8147(2) | 5848(6) | 9657(3) | 24(1) |
| C(3) | 6981(2) | 702(6) | 6062(3) | 33(1) |
| C(4) | 6545(2) | 3267(6) | 5921(3) | 24(1) |
| S(1) | 9593(1) | 3140(2) | 7315(1) | 28(1) |
| O(1) | 9527(2) | 1014(5) | 7816(2) | 50(1) |
| O(2) | 10278(2) | 4370(6) | 8008(2) | 58(1) |
| N(1) | 9888(2) | 2427(8) | 6219(3) | 34(1) |
| N(2) | 8817(2) | 4955(6) | 6848(3) | 34(1) |

**Table S3.** Anisotropic displacement parameters [Å^2^ ] for ZGlyM and ZGlyS. The anisotropic displacement factor exponent takes the form: −2π^2^ [*h*^2^ *(a*)*^2^*U*_11_*+ k*^2^ *(b*)*^2^*U*_22_ + … + *2hka*b*U*_12_ ]

Zn(C_2_H_4_NO_2_)_2_(SO_2_CH_3_NH_2_)_2_

| **Atom** | ***U*_11_** | ***U*_22_** | ***U*_33_** | ***U*_23_** | ***U*_13_** | ***U*_12_** |
| --- | --- | --- | --- | --- | --- | --- |
| Zn(1) | 40(1) | 25(1) | 24(1) | 0(1) | -4(1) | 4(1) |
| C(2) | 36(1) | 31(1) | 32(1) | -3(1) | 2(1) | -2(1) |
| N(2) | 37(1) | 26(1) | 29(1) | -1(1) | 4(1) | 2(1) |
| Zn(2) | 40(1) | 25(1) | 24(1) | 0(1) | -4(1) | 4(1) |
| C(3) | 38(1) | 38(1) | 26(1) | 2(1) | 3(1) | 7(1) |
| O(3) | 53(1) | 54(1) | 44(1) | -22(1) | -9(1) | 6(1) |
| O(4) | 58(1) | 29(1) | 45(1) | -9(1) | -17(1) | 13(1) |
| N(1) | 68(1) | 23(1) | 43(1) | -1(1) | -3(1) | 1(1) |
| C(1) | 53(1) | 59(2) | 37(1) | 4(1) | 13(1) | -6(1) |
| O(1) | 58(1) | 53(1) | 54(1) | 7(1) | -24(1) | -6(1) |
| S(1) | 41(1) | 26(1) | 26(1) | 3(1) | -3(1) | -4(1) |
| O(2) | 50(1) | 24(1) | 46(1) | 4(1) | 15(1) | -2(1) |

Zn(C_2_H_4_NO_2_)_2_[SO_2_(NH_2_)_2_]

| **Atom** | ***U*_11_** | ***U*_22_** | ***U*_33_** | ***U*_23_** | ***U*_13_** | ***U*_12_** |
| --- | --- | --- | --- | --- | --- | --- |
| Zn(1) | 35(1) | 27(1) | 16(1) | 1(1) | 6(1) | 6(1) |
| O(3) | 39(1) | 28(1) | 19(1) | -2(1) | 9(1) | -12(1) |
| O(4) | 31(1) | 37(1) | 17(1) | -5(1) | 6(1) | -3(1) |
| O(5) | 40(1) | 29(1) | 20(1) | 2(1) | 5(1) | 13(1) |
| O(6) | 56(2) | 53(2) | 19(1) | 6(1) | 3(1) | 15(1) |
| N(3) | 28(2) | 38(2) | 28(2) | -3(1) | 4(1) | -9(1) |
| N(4) | 37(2) | 26(1) | 24(1) | 1(1) | 4(1) | 7(1) |
| C(1) | 26(1) | 47(2) | 23(2) | 1(2) | 6(1) | -6(2) |
| C(2) | 22(2) | 25(2) | 23(2) | 1(1) | 3(1) | 2(1) |
| C(3) | 46(2) | 32(2) | 19(2) | -1(2) | 5(2) | 9(2) |
| C(4) | 24(2) | 30(2) | 19(2) | 2(1) | 3(1) | 0(1) |
| S(1) | 33(1) | 31(1) | 21(1) | 0(1) | 4(1) | 5(1) |
| O(1) | 59(2) | 45(2) | 52(2) | 27(2) | 25(2) | 13(1) |
| O(2) | 54(2) | 62(2) | 48(2) | -23(2) | -14(2) | 3(2) |
| N(1) | 29(2) | 43(2) | 31(2) | -8(2) | 9(1) | 3(2) |
| N(2) | 43(2) | 33(2) | 30(2) | 9(2) | 16(2) | 13(2) |

**Table S4.** Bond lengths and angles for ZGlyM and ZGlyS.

Zn(C_2_H_4_NO_2_)_2_(SO_2_CH_3_NH_2_)_2_

| Atom–Atom | Length [Å] | Atom–Atom–  Atom | Angle [°] | Atom–Atom–  Atom | Angle [°] |
| --- | --- | --- | --- | --- | --- |
| Zn(1)-N(2)#1 | 2.0128(17) | N(2)#1-Zn(1)-N(2) | 180 | Zn(2)#1-Zn(2)-O(4)#1 | 76.2(3) |
| Zn(1)-N(2) | 2.0128(17) | N(2)#1-Zn(1)-O(4)#1 | 82.39(6) | O(3)#2-Zn(2)-O(4)#1 | 101.9(2) |
| Zn(1)-O(4) | 2.0877(15) | N(2)-Zn(1)-O(4)#1 | 97.61(6) | O(3)#2-Zn(2)-O(4) | 101.6(3) |
| Zn(1)-O(4)#1 | 2.0877(15) | N(2)#1-Zn(1)-O(4) | 97.61(6) | O(4)-Zn(2)-O(4)#1 | 156.5(5) |
| C(2)-C(3) | 1.513(3) | N(2)-Zn(1)-O(4) | 82.39(6) | C(2)-C(3)-H(3A) | 109.1 |
| C(2)-O(3) | 1.258(2) | O(4)-Zn(1)-O(4)#1 | 180 | C(2)-C(3)-H(3B) | 109.1 |
| C(2)-O(4) | 1.248(2) | O(3)-C(2)-C(3) | 116.92(18) | N(2)-C(3)-C(2) | 112.65(16) |
| N(2)-H(2A) | 0.84(3) | O(4)-C(2)-C(3) | 118.73(18) | N(2)-C(3)-H(3A) | 109.1 |
| N(2)-H(2B) | 0.87(3) | O(4)-C(2)-O(3) | 124.3(2) | N(2)-C(3)-H(3B) | 109.1 |
| N(2)-Zn(2) | 2.053(3) | Zn(1)-N(2)-H(2A) | 107.6(18) | H(3A)-C(3)-H(3B) | 107.8 |
| N(2)-Zn(2)#1 | 2.065(3) | Zn(1)-N(2)-H(2B) | 111.0(19) | C(2)-O(3)-Zn(2)#3 | 125.44(14) |
| N(2)-C(3) | 1.472(3) | H(2A)-N(2)-H(2B) | 102(3) | C(2)-O(4)-Zn(1) | 113.85(13) |
| Zn(2)-N(2)#1 | 2.065(3) | Zn(2)-N(2)-H(2A) | 97.7(18) | C(2)-O(4)-Zn(2) | 111.79(14) |
| Zn(2)-Zn(2)#1 | 0.869(19) | Zn(2)#1-N(2)-H(2A) | 117.1(18) | C(2)-O(4)-Zn(2)#1 | 114.84(13) |
| Zn(2)-O(3)#2 | 2.093(10) | Zn(2)#1-N(2)-H(2B) | 100.7(19) | Zn(2)-O(4)-Zn(2)#1 | 23.5(5) |
| Zn(2)-O(4) | 2.117(2) | Zn(2)-N(2)-H(2B) | 121.2(19) | H(1A)-N(1)-H(1B) | 110(3) |
| Zn(2)-O(4)#1 | 2.148(3) | Zn(2)-N(2)-Zn(2)#1 | 24.4(5) | S(1)-N(1)-H(1A) | 112.0(19) |
| C(3)-H(3A) | 0.97 | C(3)-N(2)-Zn(1) | 111.45(13) | S(1)-N(1)-H(1B) | 118.1(19) |
| C(3)-H(3B) | 0.97 | C(3)-N(2)-H(2A) | 112.5(18) | H(1C)-C(1)-H(1D) | 109.5 |
| O(3)-Zn(2)#3 | 2.093(10) | C(3)-N(2)-H(2B) | 112.0(19) | H(1C)-C(1)-H(1E) | 109.5 |
| O(4)-Zn(2)#1 | 2.148(3) | C(3)-N(2)-Zn(2)#1 | 111.65(13) | H(1D)-C(1)-H(1E) | 109.5 |
| N(1)-H(1A) | 0.842(17) | C(3)-N(2)-Zn(2) | 110.23(14) | S(1)-C(1)-H(1C) | 109.5 |
| N(1)-H(1B) | 0.846(17) | N(2)-Zn(2)-N(2)#1 | 155.6(5) | S(1)-C(1)-H(1D) | 109.5 |
| N(1)-S(1) | 1.597(2) | N(2)-Zn(2)-O(3)#2 | 102.3(3) | S(1)-C(1)-H(1E) | 109.5 |
| C(1)-H(1C) | 0.96 | N(2)#1-Zn(2)-O(3)#2 | 102.0(3) | N(1)-S(1)-C(1) | 108.35(12) |
| C(1)-H(1D) | 0.96 | N(2)#1-Zn(2)-O(4) | 95.11(13) | O(1)-S(1)-N(1) | 108.06(11) |
| C(1)-H(1E) | 0.96 | N(2)#1-Zn(2)-O(4)#1 | 79.73(12) | O(1)-S(1)-C(1) | 109.26(13) |
| C(1)-S(1) | 1.746(2) | N(2)-Zn(2)-O(4)#1 | 94.50(14) | O(1)-S(1)-O(2) | 117.91(10) |
| O(1)-S(1) | 1.4269(18) | N(2)-Zn(2)-O(4) | 80.73(11) | O(2)-S(1)-N(1) | 105.77(11) |
| S(1)-O(2) | 1.4357(15) | Zn(2)#1-Zn(2)-N(2) | 78.6(3) | O(2)-S(1)-C(1) | 107.14(11) |
|  |  | Zn(2)#1-Zn(2)-N(2)#1 | 77.1(3) |  |  |
|  |  | Zn(2)#1-Zn(2)-O(3)#2 | 178.00(15) |  |  |
|  |  | Zn(2)#1-Zn(2)-O(4) | 80.3(3) |  |  |

Symmetry transformations used to generate equivalent atoms:

#1 -x+1/2,-y+1/2,-z #2 -x+1/2,y-1/2,-z+1/2 #3 -x+1/2,y+1/2,-z+1/2

Zn(C_2_H_4_NO_2_)_2_[SO_2_(NH_2_)_2_]

| Atom–Atom | Length [Å] | Atom–Atom–  Atom | Angle [°] | Atom–Atom–  Atom | Angle [°] |
| --- | --- | --- | --- | --- | --- |
| Zn(1)-O(3) | 2.096(2) | O(4)-Zn(1)-O(3)#1 | 100.43(9) | C(2)-C(1)-H(1A) | 109.3 |
| Zn(1)-O(4) | 2.035(2) | O(4)-Zn(1)-O(5) | 94.84(9) | C(2)-C(1)-H(1B) | 109.3 |
| Zn(1)-O(5) | 2.077(2) | O(4)-Zn(1)-N(3)#1 | 105.99(11) | O(3)-C(2)-O(4) | 124.8(3) |
| Zn(1)-N(3) | 2.055(3) | O(4)-Zn(1)-N(4) | 103.48(10) | O(3)-C(2)-C(1) | 119.6(3) |
| Zn(1)-N(4) | 2.065(3) | O(5)-Zn(1)-O(3)#1 | 164.56(9) | O(4)-C(2)-C(1) | 115.6(3) |
| O(3)-C(2) | 1.242(4) | N(3)#1-Zn(1)-O(3)#1 | 81.04(10) | N(4)-C(3)-H(3C) | 109.0 |
| O(4)-C(2) | 1.273(4) | N(3)#1-Zn(1)-O(5) | 97.00(10) | N(4)-C(3)-H(3D) | 109.0 |
| O(5)-C(4) | 1.270(4) | N(3)#1-Zn(1)-N(4) | 150.51(12) | N(4)-C(3)-C(4) | 112.7(3) |
| O(6)-C(4) | 1.246(4) | N(4)-Zn(1)-O(3)#1 | 92.72(10) | H(3C)-C(3)-H(3D) | 107.8 |
| N(3)-H(3A) | 0.8900 | N(4)-Zn(1)-O(5) | 81.43(10) | C(4)-C(3)-H(3C) | 109.0 |
| N(3)-H(3B) | 0.8900 | C(2)-O(3)-Zn(1)#2 | 112.9(2) | C(4)-C(3)-H(3D) | 109.0 |
| N(3)-C(1) | 1.468(4) | C(2)-O(4)-Zn(1) | 120.5(2) | O(5)-C(4)-C(3) | 118.9(3) |
| N(4)-H(4A) | 0.8900 | C(4)-O(5)-Zn(1) | 114.9(2) | O(6)-C(4)-O(5) | 124.5(3) |
| N(4)-H(4B) | 0.8900 | Zn(1)#2-N(3)-H(3A) | 109.3 | O(6)-C(4)-C(3) | 116.5(3) |
| N(4)-C(3) | 1.458(4) | Zn(1)#2-N(3)-H(3B) | 109.3 | O(1)-S(1)-N(1) | 114.2(2) |
| C(1)-H(1A) | 0.9700 | H(3A)-N(3)-H(3B) | 108.0 | O(1)-S(1)-N(2) | 105.06(18) |
| C(1)-H(1B) | 0.9700 | C(1)-N(3)-Zn(1)#2 | 111.4(2) | O(2)-S(1)-O(1) | 114.36(19) |
| C(1)-C(2) | 1.512(5) | C(1)-N(3)-H(3A) | 109.3 | O(2)-S(1)-N(1) | 106.2(2) |
| C(3)-H(3C) | 0.9700 | C(1)-N(3)-H(3B) | 109.3 | O(2)-S(1)-N(2) | 115.40(18) |
| C(3)-H(3D) | 0.9700 | Zn(1)-N(4)-H(4A) | 109.3 | N(1)-S(1)-N(2) | 101.08(19) |
| C(3)-C(4) | 1.514(4) | Zn(1)-N(4)-H(4B) | 109.3 | S(1)-N(1)-H(1C) | 117(3) |
| S(1)-O(1) | 1.442(3) | H(4A)-N(4)-H(4B) | 108.0 | S(1)-N(1)-H(1D) | 115(3) |
| S(1)-O(2) | 1.428(3) | C(3)-N(4)-Zn(1) | 111.4(2) | H(1C)-N(1)-H(1D) | 108(4) |
| S(1)-N(1) | 1.590(3) | C(3)-N(4)-H(4A) | 109.3 | S(1)-N(2)-H(2A) | 116(3) |
| S(1)-N(2) | 1.607(3) | C(3)-N(4)-H(4B) | 109.3 | S(1)-N(2)-H(2B) | 104(3) |
| N(1)-H(1C) | 0.81(4) | N(3)-C(1)-H(1A) | 109.3 | H(2A)-N(2)-H(2B) | 122(4) |
| N(1)-H(1D) | 0.80(4) | N(3)-C(1)-H(1B) | 109.3 |  |  |
| N(2)-H(2A) | 0.73(4) | N(3)-C(1)-C(2) | 111.6(3) |  |  |
| N(2)-H(2B) | 0.86(5) | H(1A)-C(1)-H(1B) | 108.0 |  |  |

Symmetry transformations used to generate equivalent atoms:

#1 -x+3/2,y-1/2,-z+2 #2 -x+3/2,y+1/2,-z+2

**Table S5.** Direction and magnitude of the dipole moment of ZnO_3_N_2_ and SO_2_N_2_ groups in Zn(C_2_H_4_NO_2_)_2_[SO_2_(NH_2_)_2_].

| **Species** | **D_x_** | **D_y_** | **D_z_** | **Magnitude** |
| --- | --- | --- | --- | --- |
| ZnO_3_N_2_ | -1.44538 | 0.828206 | -0.24649 | 1.683984 |
| ZnO_3_N_2_ | -1.44536 | 0.828179 | -0.24647 | 1.683954 |
| ZnO_3_N_2_ | 1.443091 | 0.828033 | 0.245724 | 1.681823 |
| ZnO_3_N_2_ | 1.443091 | 0.828033 | 0.245724 | 1.681823 |
| ∑ZnO_3_N_2_ | **-0.00456** | **3.312452** | **-0.00151** |  |

| **Species** | **D_x_** | **D_y_** | **D_z_** | **Magnitude** |
| --- | --- | --- | --- | --- |
| SO_2_N_2_ | 0.985598 | 0.969238 | 4.482613 | 4.690911 |
| SO_2_N_2_ | 0.985598 | 0.969238 | 4.482613 | 4.690911 |
| SO_2_N_2_ | -1.01685 | 0.971338 | -4.4973 | 4.712029 |
| SO_2_N_2_ | -0.9877 | 0.968831 | -4.49795 | 4.705921 |
| ∑SO_2_N_2_ | **-0.03335** | **3.878644** | **-0.03002** |  |


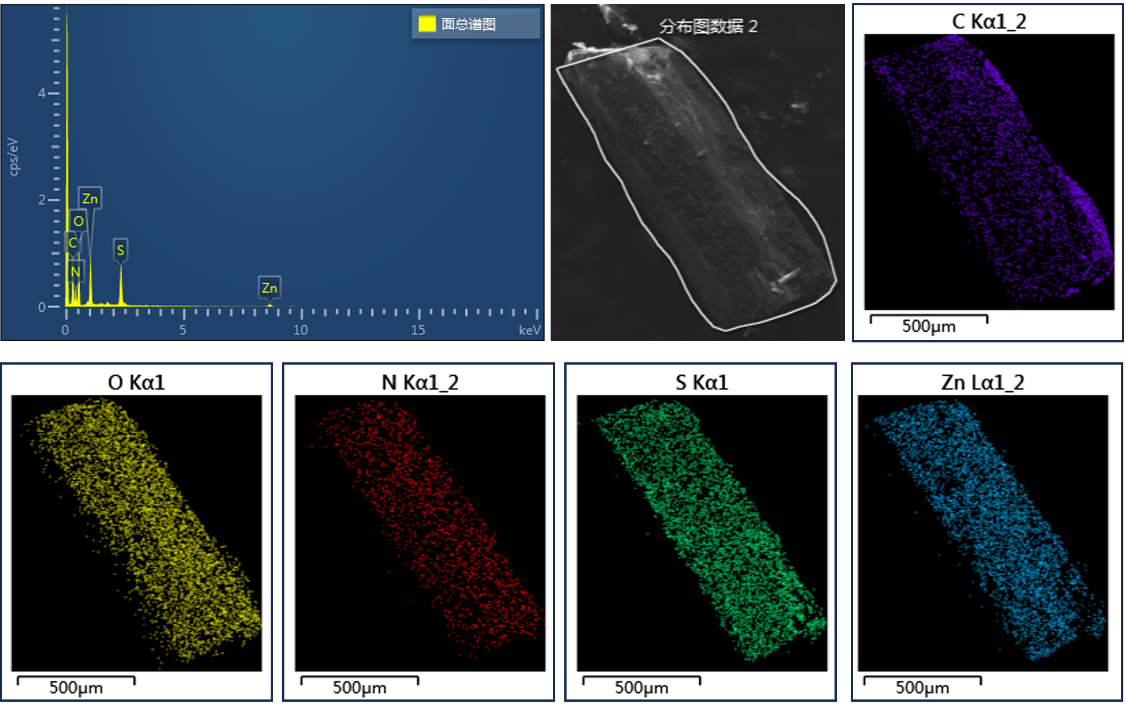


Figure S1. The scanning electron microscope elemental

mapping of ZGlyS


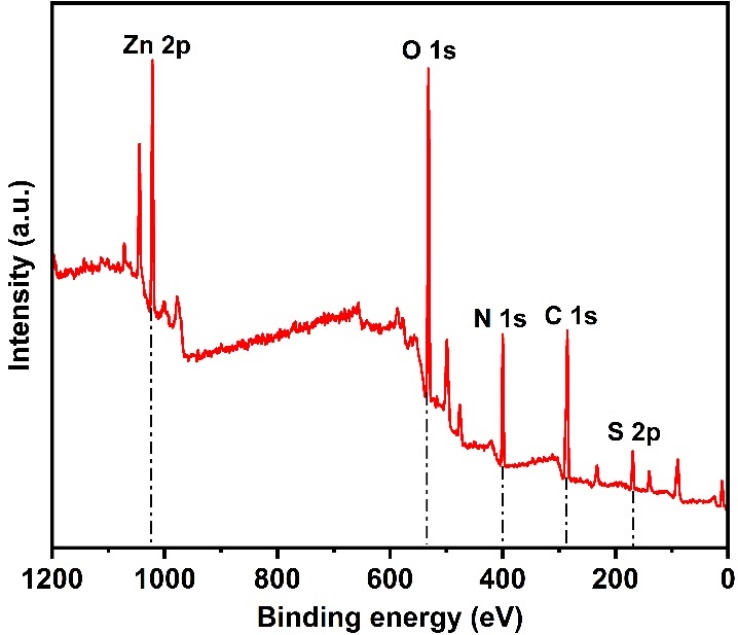


Figure S2. The XPS survey scan of ZGlyS


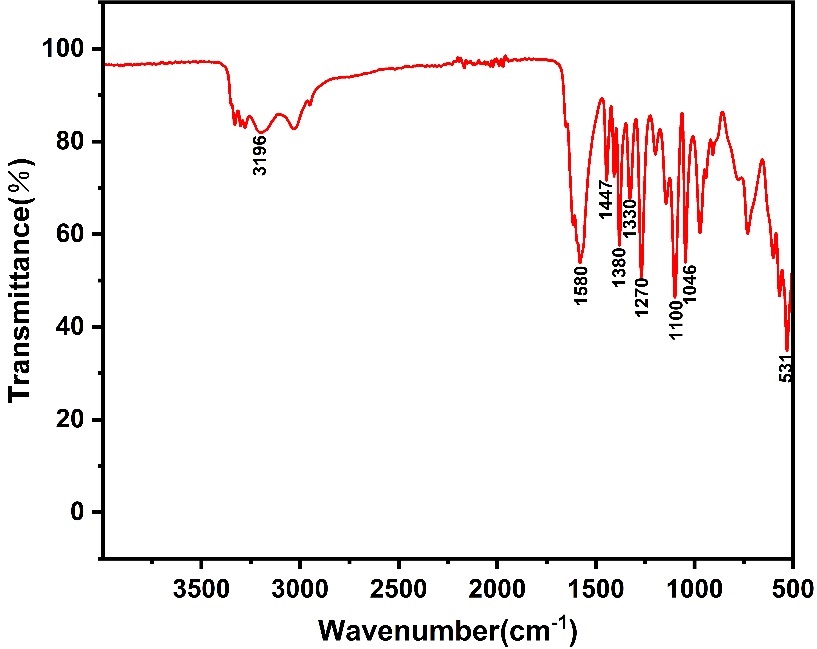


Figure S3. The IR spectrum of ZGlyS


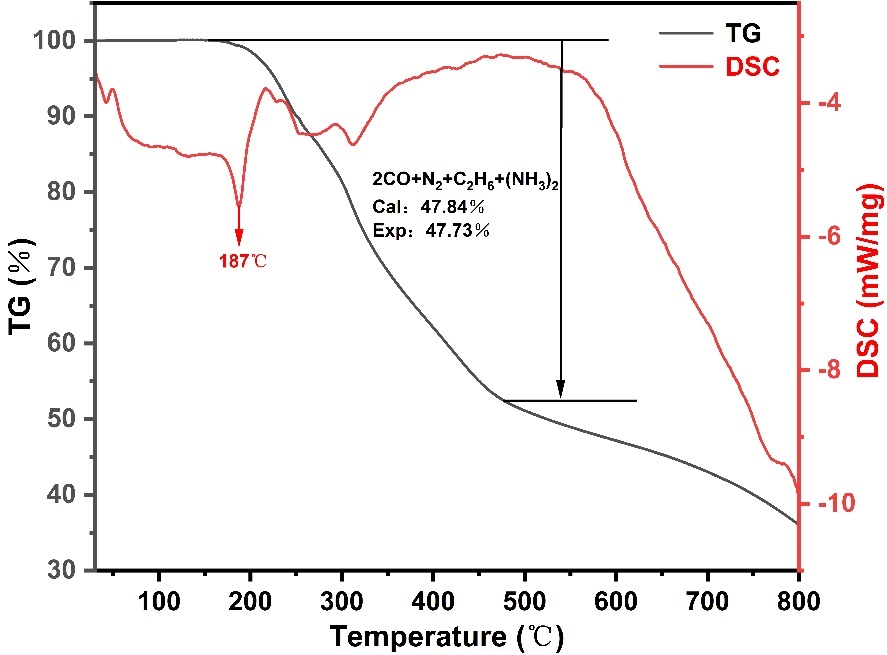


Figure S4. TG-DSC curve of ZGlyS

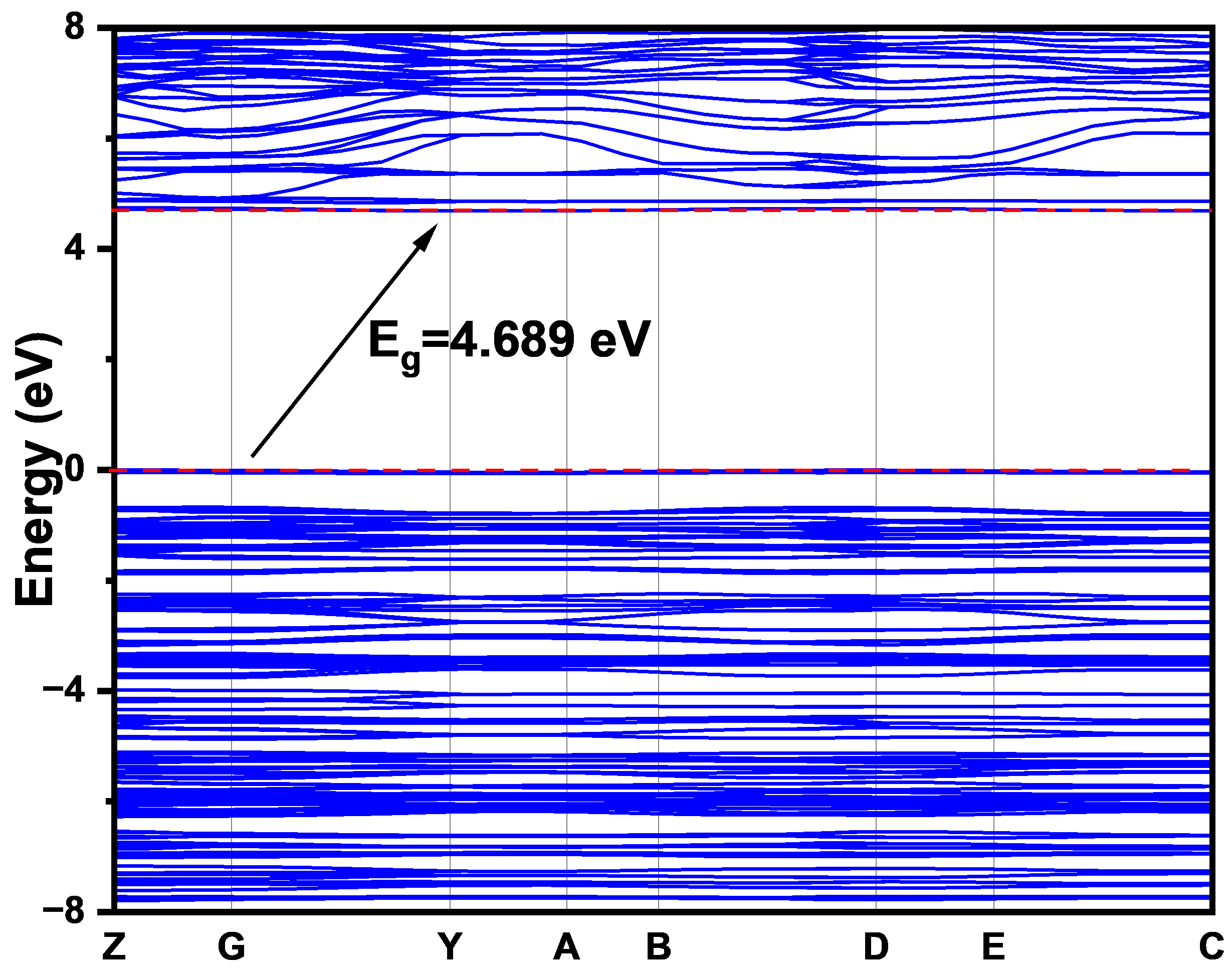


Figure S5. The calculated band structure of ZGlyS


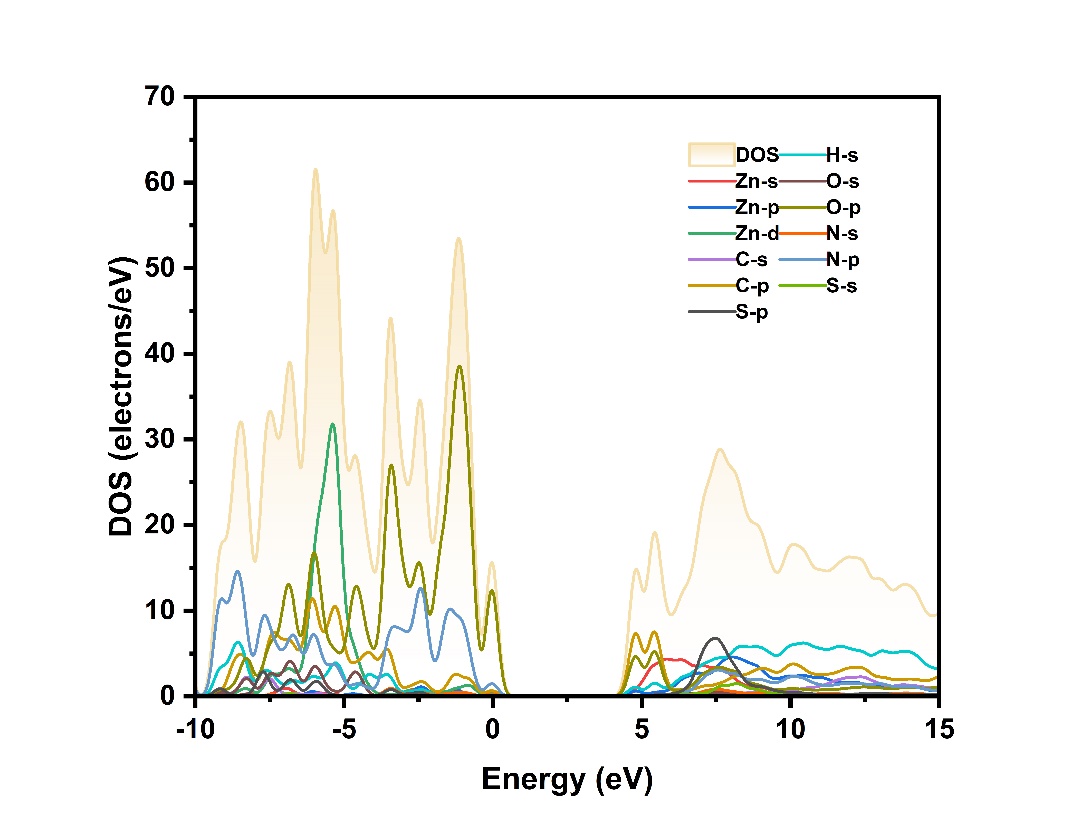


Figure S6. DOS and PDOS of ZGlyS

**Table S6.** Performance comparison of main Gly-containing compounds

| Formula | Space group | Band Gap(eV) | SHG (× KDP) | Birefringence | No./Ref. |
| --- | --- | --- | --- | --- | --- |
| NaF·C_2_H_5_NO_2_ | N/A | 5.81 | 0.59 | N/A | 1#/11 |
| KI·C_2_H_5_NO_2_ | *Cc* | 4.77 | 0.89 | N/A | 2#/12 |
| NaI·C_2_H_5_NO_2_ | *Pm* | 5.09 | 1.53 | N/A | 3#/13 |
| LiCl·C_2_H_5_NO_2_ | N/A | 5.53 | 3.00 | N/A | 4#/14 |
| ZnCl_2_·C_2_H_5_NO_2_ | N/A | 5.16 | 0.50 | N/A | 5#/15 |
| SbF_3_·C_2_H_5_NO_2_ | *Pc* | 4.63 | 3.60 | 0.057@1064 nm | 6#/16 |
| *α*-2SbF_3_·C_2_H_5_NO_2_ | *Ia* | 4.78 | 3.30 | 0.146@532 nm | 7*#*/17 |
| CaBr_2_·(C_2_H_5_NO_2_)_3_ | *Pbc*2_1_ | 5.30 | 1.20 | N/A | 8#/18 |
| CaCl_2_·(C_2_H_5_NO_2_)_3_ | *Pb*2_1_*a* | 4.46 | 1.50 | N/A | 9#/19 |
| ZnCl_2_·(C_2_H_5_NO_2_)_3_ | *Pbn*2_1_ | 5.08 | 0.50 | N/A | 10#/20 |
| NaNO_3_·C_2_H_5_NO_2_ | *Cc* | 5.26 | 1.86 | N/A | 11#/21 |
| NaNO_2_·(C_2_H_5_NO_2_)_3_ | N/A | 6.31 | 1.27 | N/A | 12#/22 |
| Ba(NO_3_)_2_·C_2_H_5_NO_2_ | *P*2_1_2_1_2_1_ | 5.90 | 0.80 | N/A | 13#/23 |
| Li_2_SO_4_·C_2_H_5_NO_2_ | *Pna*2_1_ | 5.20 | 0.70 | 0.144@550 nm | 14#/24 |
| LiNO_3_·(C_2_H_5_NO_2_)_2_ | *Pca*2 | 5.17 | 1.50 | N/A | 15#/25 |
| H_2_SO_4_·(C_2_H_5_NO_2_)_3_ | *P*2_1_ | 5.44 | 0.59 | N/A | 16#/26 |
| (NH_4_)_2_SO_4_·(C_2_H_5_NO_2_)_2_ | *P*3_2_ | 5.22 | 1.45 | N/A | 17#/27 |
| Na_2_MoO_4_·(C_2_H_5_NO_2_)_3_ | N/A | 5.07 | 0.74 | N/A | 18#/28 |
| ZnSO_4_ C_2_H_5_NO_2_·3H_2_O | *Pca*2_1_ | 4.96 | 2.00 | N/A | 19#/29 |
| ZnSO_4_·C_2_H_5_NO_2_·7H_2_O | N/A | 4.13 | 0.70 | N/A | 20#/30 |
| ZnSO_4_·C_2_H_4_NO_2_·Na_2_SO_4_·7H_2_O | N/A | 5.85 | 0.62 | N/A | 21#/31 |
| (HC_2_H_5_NO_2_)_2_·Zn(SO_4_)(C_2_O_4_) | *C*2 | 4.70 | 0.30 | 0.030@1064 nm | 22#/32 |
| HC_2_H_5_NO_2_·In(SO_4_)(C_2_O_4_)(C_2_H_5_NO_2_) | *P*2_1_ | 4.60 | 0.90 | 0.020@1064 nm | 23#/32 |
| (HNO_3_)_0.5_·(C_2_H_5_NO_2_)·(H_2_SO_4_)_0.5_ | N/A | 5.29 | 0.03 | N/A | 24#/33 |
| Zn(C_2_H_4_NO_2_)_2_·[SO_2_(NH_2_)_2_] | *C*2 | 5.93 | 1.36 | 0.126@546 nm | 25#/This work |
| (C_2_H_5_NO_2_)_2_·HClO_4_ | *P*$\bar{1}$ | 6.16 | / | N/A | 34 |
| *β*-2SbF_3_·C_2_H_5_NO_2_ | *P*2_1_/*c* | 4.41 | / | 0.070@532 nm | 17 |
| SrBr_2_·(C_2_H_5_NO_2_)_2_·3H_2_O | *Pbcn* | 4.56 | / | N/A | 35 |
| LiClO_4_·(C_2_H_5_NO_2_)_2_ | *P*2_1_/*c* | 5.70 | / | N/A | 36 |
| [Zn(H_2_O)_6_][Zn(C_2_H_5_NO_2_)_2_(H_2_O)_4_](SO_4_)_2_ | *P*$\bar{1}$ | 5.12 | / | N/A | 37 |
| HF·C_2_H_5_NO_2_ | *Pbca* | 5.34 | (0.60) | N/A | 38 |
| ZnCl_2_·(C_2_H_5_NO_2_)_2_·H_2_O | *C*2/*c* | 2.20 | (0.88) | N/A | 39 |
| BaCl_2_·(C_2_H_5_NO_2_)_3_ | *Pbcn* | 5.40 | (0.50) | N/A | 40 |
| BaCl_2_·(C_2_H_5_NO_2_)_2_·H_2_O | *Pbcn* | 5.16 | (2.18) | N/A | 41 |
| Zn_2_(C_2_H_4_NO_2_)_4_·2H_2_O | *P*$\bar{1}$ | 5.14 | (1.61) | N/A | 42 |

N/A: Not available. The lower part of the table lists some crystals with centrosymmetric structures, but they have been reported to exhibit SHG effects (marked in parentheses). We believe it goes against common sense.

**Reference**

1. V. Dolomanov, L. J. Bourhis, R. J. Gildea, J. A. K. Howard, H. Puschmann. *J. Appl. Crystallogr.* **2009**, *42*, 339-341.
2. L. Spek, J. Appl. *Crystallogr.* **2003**, *36*, 7-13.
3. S. K. Kurtz, T. T. Perry. *J. Appl. Phys.* **1968**, *39*, 3798-3813.
4. B. E. Sørensen, *Eur. J. Mineral.* **2013**, *25*, 5-10.
5. M. C. Payne, M. P. Teter, D. C. Allan, T. A. Arias, J. D. Joannopoulos, *Rev. Mod. Phys.* **1992,** *64 (4)*, 1045-1097.
6. S. J. Clark, M. D. Segall, C. J. Pickard, P. J. Hasnip, M. J. Probert, K. Refson, M. C. Payne, *Z. Krist.* **2005**, *220,* 567-570.
7. A. M. Rappe, K. M. Rabe, E. Kaxiras, J. D. Joannopoulos, *Phys. Rev. B* **1990**, *41*, 1227-1230.
8. L. Kleinman, D. M. Bylander, *Phys. Rev. Lett.* **1982,** *48* *(20)*, 1425-1428.
9. C. S. Wang, B. M. Klein, *Phys. Rev. B* **1981,** *24* *(6)*, 3393-3416.
10. R. W. Godby, M. Schluter, L. J. Sham, *Phys. Rev. B* **1988,** *37* *(17)*, 10159-10175.
11. K. Suresh, P. Jayaprakash, K. Thillaivelavan, R. Ravisankar. *J. Mater. Sci-mater EI* **2023**, *34*,1986.
12. P. Jayaprakash, P. Krishnan, K. Suresh, K. Thillaivelavan, B. Dhinakaran, G. Vinitha, R. Ravisankar. *Chem. Data Collect* **2021**, *34*, 100752.
13. B. Neelakantaprasad, G. Rajarajan, B. Ravi, S. Masilamani. *J. Mater. Sci-mater EI* **2018,** *29*, 20225-20235.
14. M. Lenin, G. Bhavannarayana, P. Ramasamy. *Opt. Commun.* **2009**, *282*, 1202-1206.
15. T. Balakrishnan, K. Ramamurthi. *Mater. Lett.* **2008**, *62*, 65-68.
16. Z. Bai, J. Lee, C. Hu, G. Zou, K. M. Ok. *Chem. Sci.* **2024**, *15*, 6572-6576.
17. Y. Kuk, Z. Bai, Y. Li, K. M. Ok. *Chem. Mater.* **2025**, *37*, 1306-1313.
18. G. Babu Rao, P. Rajesh, P. Ramasamy. *J. Cryst. Growth* **2016**, *440*,47-54.
19. P.V. Dhanaraj, N. P. Rajesh. *PHYSICA B* **2011**, *406*, 12-18.
20. M. Esthaku Peter, P. Ramasamy. *Mater. Lett.* **2010**, *64*, 1-3.
21. R. Ravisankar1, P. Jayaprakash, P. Eswaran, K. Mohanraj, G. Vinitha, M. Pichumani. *J. Mater. Sci-mater EI* **2020**, *31*, 17320-17331.
22. M. M. Khandpekar, S. P. Pati. *Opt. Commun.* **2012**, *285*, 288-293.
23. S. Varalakshmi, S. M. Ravi Kumar, G. Elango, R. Ravisankar. *Spectrochim. Acta, Part A* **2014**, *133*, 677-682.
24. Z. Bai, S. Chen, X. Chen, Y. Wang, X. Zhou, Y. Song, Y. Li, S. Zhao, J. Luo. *J. Mater. Chem. C* **2025**, *13*, 19408-19416.
25. J. Dalal, N. Sinha, B. Kumar. *Opt. Mater.* **2014**, *37*, 457-463.
26. A. Parameswari, R. Mohamed Asath, R. Premkumar, A. Milton Franklin Benial. *J. Mol. Struct.* **2017**, *1128*, 428-438.
27. B. Helina. *Optik* **2016**, *127*, 1401-1404.
28. K. Amarsingh Bhabu, S. R. Balaji, R. Sree Devi, R. Sree Devi, T. Balu, G. Muralidharand, T.R. Rajasekarana, *Optik* **2016**, *127*, 1708-1713.
29. R. Sankar, C. M. Raghavan, M. Balaji, R. Mohan Kumar, R. Jayavel. *Cryst. Growth Des.* **2007**, *7 (2)*, 348-353.
30. T. Balakrishnan, K. Ramamurthi. *Spectrochim. Acta, Part A* **2007**, *68*, 360-363.
31. M. M. Khandpekar, S. S. Dongare, S.B. Patil, S. P. Pati. *Opt. Commun.* **2011**, *284*, 3548-3551.
32. Y. Li, M. Luo, Y. Long, L. Huang, D. Gao, G. Zou, Z. Lin, Y. Zhao. *Inorg. Chem.* **2023**, *62*, 8500-8504.
33. M. M. Khandpekar, S. P. Pati. *Opt. Commun.* **2010**, *283*, 2700-2704.
34. L. Panicker, P. Mathur, S. M. Mobin. *J. Chem. Crystallogr*. **2011**, *41*,147-154.
35. P. Revathi, M. George, B. M. Philip, S. S. Nair, R. Anjana, B. K. Jose, D. S. Manoj, T. Balakrishnan, D. Sajan. *J. Mater. Sci-mater EI* **2024**, *35*, 842.
36. P. Revathi, M. George, B. M. Philip, S. S. Nair, B. K. Jose, E. Shereena, R. Ittyachan, T. Balakrishnan, D. Sajan. *Cryst. Res. Technol.* **2024**, *59*, 2300097.
37. Kiran, N. Vijayan, D. Nayak, M. Kumari, Vinod, K. Kumar, P. Vashishtha, N. Thirughanasambantham, V. Balachandran, B. Sridhar, G. Gupta. *J. Mater. Sci-mater EI* **2023**, *34*, 1132.
38. A. Abu El-Fadl, A. M. Abdel-Salam, A. M. Nashaat. *Mater Sci-Poland* **2018**, *36 (4)*, 685-696.
39. B. Uma, Rajnikant, K. S. Murugesan, S. Krishnan, B. M. Boaz. *Prog. Nat. Sci-Mater* **2014**, *24*, 378-387.
40. S. Chennakrishnan, S. M. Ravi Kumar, D. Sivavishnu, M. Ganapathi, I. Vetha Potheher, M. Vimalan. *J. Mater. Sci-mater EI* **2016**, *27*, 10113-10121.
41. G. Marudhu, S. Krishnan, T. Thilak, P. Samuel, G. Vinitha, G. Pasupathi. *J. Nonlinear Opt. Phys.* **2013**, *22 (4)*, 1350043.
42. K. C. Bright, R. N. Anila, B. Anugop, M. Kailasnath. *J. Mater. Sci-mater EI* **2024**, *35*, 2224.
